# Lipid derivatives activate GPR119 and trigger GLP-1 secretion in primary murine L-cells

**DOI:** 10.1016/j.peptides.2015.06.012

**Published:** 2016-03

**Authors:** Daryl Hodge, Leslie L. Glass, Eleftheria Diakogiannaki, Ramona Pais, Carol Lenaghan, David M. Smith, Marianne Wedin, Mohammad Bohlooly-Y, Fiona M. Gribble, Frank Reimann

**Affiliations:** a Metabolic Research Laboratories and MRC Metabolic Diseases Unit, WT-MRC Institute of Metabolic Science, Addenbrooke’s Hospital, Cambridge CB2 0QQ, UK; b AstraZeneca, Cardiovascular & Metabolic Diseases iMed, Alderley Park, Cheshire, UK; c AstraZeneca, Cardiovascular & Metabolic Diseases iMed, Mölndal, Sweden; d AstraZeneca, Transgenics Group, Reagents & Assay Development, Discovery Sciences, Mölndal, Sweden

**Keywords:** 2-OG, 2-oleoylglycerol, Fsk, forskolin, GLP-1, glucagon-like peptide-1, IBMX, 3-isobutyl-1-methylxanthine, KO, knockout, OEA, oleoylethanolamide, PPAR, peroxisome-proliferator-activated receptor, WT, wildtype, GLP-1, GPR119, Incretin

## Abstract

**Aims/hypothesis:**

Glucagon-like peptide-1 (GLP-1) is an incretin hormone derived from proglucagon, which is released from intestinal L-cells and increases insulin secretion in a glucose dependent manner. GPR119 is a lipid derivative receptor present in L-cells, believed to play a role in the detection of dietary fat. This study aimed to characterize the responses of primary murine L-cells to GPR119 agonism and assess the importance of GPR119 for the detection of ingested lipid.

**Methods:**

GLP-1 secretion was measured from murine primary cell cultures stimulated with a panel of GPR119 ligands. Plasma GLP-1 levels were measured in mice lacking GPR119 in proglucagon-expressing cells and controls after lipid gavage. Intracellular cAMP responses to GPR119 agonists were measured in single primary L-cells using transgenic mice expressing a cAMP FRET sensor driven by the proglucagon promoter.

**Results:**

L-cell specific knockout of GPR119 dramatically decreased plasma GLP-1 levels after a lipid gavage. GPR119 ligands triggered GLP-1 secretion in a GPR119 dependent manner in primary epithelial cultures from the colon, but were less effective in the upper small intestine. GPR119 agonists elevated cAMP in ∼70% of colonic L-cells and 50% of small intestinal L-cells.

**Conclusions/interpretation:**

GPR119 ligands strongly enhanced GLP-1 release from colonic cultures, reflecting the high proportion of colonic L-cells that exhibited cAMP responses to GPR119 agonists. Less GPR119-dependence could be demonstrated in the upper small intestine. In vivo, GPR119 in L-cells plays a key role in oral lipid-triggered GLP-1 secretion.

## Highlights

• GPR119, a putative fat sensor, is a potential target for metabolic disease.
• KO of GPR119 in murine L-cells reduced GLP-1 response to fat in vivo.
• Primary L-cells secreted GLP-1 in response to GPR119 agonists.
• GPR119 agonists increased L-cell cAMP, with greatest efficacy in the colon.
• Our data support the use of GPR119 agonists to raise GLP-1 levels.

## 1 Introduction

Glucagon-like peptide-1 (GLP-1) has multiple anti-diabetic effects, most notably enhancing insulin secretion, suppressing glucagon release and slowing gastric emptying [1]. Current incretin-based therapies focus on preventing the breakdown of GLP-1 by dipeptidyl peptidase-IV or administrating GLP-1 mimetics [2]. The benefits of increasing endogenous GLP-1 secretion are currently under evaluation, supported by evidence that gastric bypass surgery improves glucose tolerance, at least in part by increased GLP-1 secretion [3].

GPR119 is one of a number of candidate G-protein coupled receptors currently under investigation as a potential target for elevating GLP-1 and insulin release. GLP-1 is secreted from enteroendocrine L-cells in the intestinal epithelium, which express a variety of receptors and transporters capable of detecting ingested nutrients, including carbohydrates, lipids and proteins [4]. GPR119 is a Gα_s_-coupled receptor, linked to the elevation of intracellular cAMP concentrations [5], [6], [7], [8], [9], [10], [11]. Physiological GPR119 ligands include oleoylethanolamide (OEA) [6], produced locally within tissues [12], [13], [14], and 2-oleoyl glycerol (2-OG) [15] generated by luminal triacylglycerol digestion [16]. OEA as well as small molecule GPR119 agonists, increase GLP-1 and insulin release in rodent models [9], [17], [18], [19]. Indeed, GPR119 agonists were developed for human studies and taken into clinical trials in patients with type 2 diabetes, but were not found to improve metabolic control [20]. The reasons for the poor translatability remain uncertain, and the physiological roles and therapeutic potential of GPR119 are still under investigation.

The aim of this study was to investigate the physiological role of GPR119, and the signaling events triggered by GPR119 agonists in native murine L-cells. Using a fluorescent reporter providing a readout of cAMP concentrations in living native L-cells, we show that OEA, 2-OG, and a specific GPR119 agonist elevated cytoplasmic cAMP concentrations and enhanced GLP-1 secretion in primary cultured L-cells. We further present a new conditional knockout (KO) mouse model lacking GPR119 in proglucagon expressing cell populations including L-cells and alpha-cells. Oral oil tolerance tests in wild type (WT) and KO mice revealed that lipid-triggered plasma GLP-1 excursions are highly dependent on activation of GPR119 in L-cells.

## 2 Methods

### 2.1 Animal models

The flox *Gpr119* mouse (*Gpr119^fl^*) was created using the embryonic stem cell method by AstraZeneca Transgenics and Comparative Genetics, Mölndal, Sweden. Genotyping for *Gpr119^fl^* was performed using the primers: Forward, TGCAGAGAGGGAGCAAATATCAGG; Reverse, TCTTGTTGTAACAAGCCTTCCAGG. Conditional *Gpr119* knockout mice were created by crossing homozygous *Gpr119^fl^* with heterozygous GLUCre12 mice, which express *Cre recombinase* under proglucagon promoter control [21]. The mice were selectively bred to produce homozygous females or hemizygous males (*Gpr119* is located on the X-chromosome) for *Gpr119^fl^*. All mice were on a C57BL/6 background. Details of generation of Glu-Epac21 mice are described elsewhere [22]. Briefly, this is a transgenic strain in which the cAMP FRET sensor, Epac2-camps, is expressed under control of the mouse glucagon receptor, using the same starting BAC and technique as used previously to generate GLU-Venus mice [23]. The L-cell-specificity of Epac2-camps expression was confirmed by immunofluorescence staining of fixed intestinal tissue slices. Mice were kept in individually ventilated cages according to UK Home Office regulations and the ‘Principles of laboratory animal care.’ All procedures were approved by a local ethical review committee.

### 2.2 Primary murine intestinal cell culture

Mice aged six weeks to six months were killed by cervical dislocation. Intestines were collected into ice-cold Leibovitz’s L-15 medium (PAA, Yeovil, UK) and primary intestinal culture performed was as previously described [23]. Duodenum/jejunum was taken as a 10 cm length distal to the pylorus; 10 cm of ileum was taken proximal to the ileocecal junction, and colon included all tissue distal to the caecum. Minced tissue was digested with 0.4 mg/ml collagenase XI in Dulbecco’s Modified Eagle Medium (DMEM) containing 4.5 g/l glucose. Crypts were pelleted at 100 g for 3 min before resuspension in DMEM containing 10% fetal bovine serum, 2 mmol/l L-glutamine, 100 units/ml penicillin, and 0.1 mg/mL streptomycin. 10 μmol/l of the Rho-associated, coiled-coil containing protein kinase (ROCK) inhibitor Y27632 was added to small intestinal cultures. Cells were plated onto 24-well plates (secretion) or glass-bottom dishes (imaging) coated with a 1:100 dilution of Matrigel (BD Biosciences, Oxford, UK). Each 24-well culture plate contained crypt suspensions from a single mouse. Cultures were incubated at 37 °C and 5% CO_2_.

### 2.3 Intestinal cell secretion

Secretion studies were carried out 20–24 h post-plating, as described previously [23]. Total GLP-1 concentrations were analyzed in test solutions and cell lysates by immunoassay (MesoScale Discovery, Gaithersburg, MD, USA). Hormone levels in the test solution and cell lysate were summed to give the total well content. GLP-1 secretion was expressed as a percentage of this total.

### 2.4 Lipid gastric gavage

Mixed male and female adult mice were used for the gavage study, and the groups did not differ significantly in body weight. GLP-1 levels were similar in the male and female mice, so data were combined. Mice were fasted overnight (<16 h). Intragastric gavage of a 1:1 mix of olive:corn oils was administered (10 ml/kg body weight). Control wild type mice received a gavage of phosphate buffered saline. 25 min later, mice were anaesthetized with isoflurane, and terminal blood samples taken at 30 min by cardiac puncture. Plasma was separated immediately and frozen. Total GLP-1 in the plasma was measured by immunoassay (Mesoscale Discovery).

### 2.5 cAMP Imaging

Single-cell measurements of cAMP levels were made using the Förster resonance energy transfer (FRET)-based sensor Epac2-camps, using tissues from Glu-Epac21 mice maintained in mixed primary culture for 20-78 h. The use of Epac2-camps for monitoring cAMP concentrations in GLP-1 expressing cell lines has been described previously [24]. Maximum time-averaged CFP/YFP ratios, representing [cAMP], were determined at baseline and following test reagent application.

### 2.6 Solutions

Saline buffer contained (in mmol/l: 138 NaCl, 4.5 KCl, 4.2 NaHCO_3_, 1.2 NaH_2_PO_4_, 2.6 CaCl_2_, 1.2 MgCl_2_, 10 HEPES, pH 7.4 with NaOH) supplemented with 0.1% bovine serum albumin (BSA). Solutions for secretion studies included 10 mmol/l glucose and DMSO at a final concentration of 0.1%. Unless stated, all reagents were purchased from Sigma (Poole, UK). AR231453 was synthesized by AstraZeneca.

### 2.7 Data analysis

Data were analyzed using Microsoft Excel and GraphPad Prism v5.0 (Graphpad Software, San Diego, USA), using Student’s *t*-tests, ANOVA and post-hoc Bonferroni tests, as indicated in the figure legends.

## 3 Results

### 3.1 GPR119 involvement in lipid-sensing

**Fig. 1:**
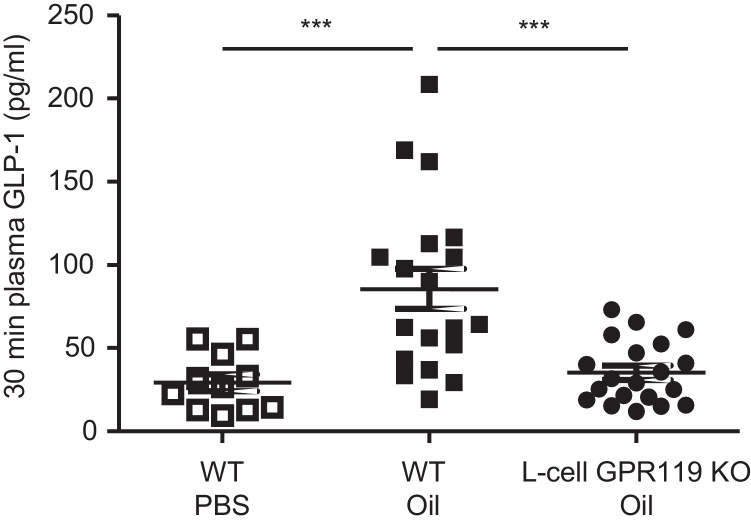
L-cell knockout of *Gpr119* impairs lipid-triggered GLP-1 release in vivo. Plasma GLP-1 (total) 30 min after gavage of 10 μl/g olive and corn oil mix (1:1) in *Gpr119* WT and KO mice, or of PBS in WT mice. Significance was tested by ANOVA with post-hoc Bonferroni test; ***p < 0.001.

The contribution of GPR119 to GLP-1 secretion in vivo was investigated by the administration of a lipid gastric gavage to *Gpr119-*KO and WT mice. In WT mice, oil gavage triggered an approximate 3-fold elevation of plasma total GLP-1 concentrations at 30 min, compared with control mice gavaged with saline (Fig. 1). GLP-1 after oil gavage was significantly lower in KO animals compared to WT controls (Fig. 1), indicating that GPR119 in L-cells plays an important role in mediating the GLP-1 secretory response to ingested triglyceride.

### 3.2 GPR119-dependent GLP-1 secretion in vitro

**Fig. 2:**
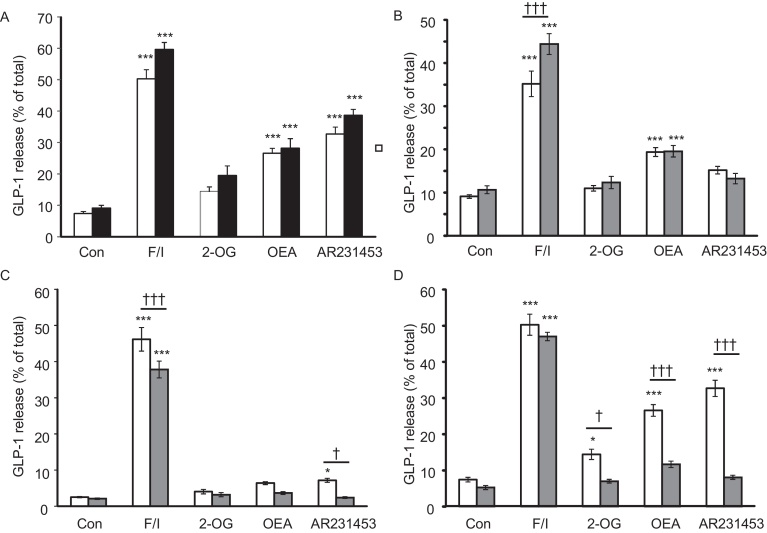
L-cell knockout of gpr119 impairs lipid-triggered GLP-1 release in primary cultures. (A) Mice carrying only the floxed *Gpr119* alleles (*Cre*-negative/*Gpr119*^fl^, white bars, *n *= 22 wells from 7 mice per column), or L-cell Cre-recombinase (*Cre*-positive/*Gpr119*^wt^ mice, black bars, *n *= 9 wells from 3 mice), did not differ in their responses to GPR119 ligands. GLP-1 secretion was measured under basal conditions (Con) and in response to 10 μmol/l forskolin + 100 μmol/l IBMX (F/I), 2-oleoylglycerol (2-OG, 200 μmol/l), oleoylethanolamide (OEA, 10 μmol/l) or AR231453 (100 nmol/l). All conditions contained 10 mM glucose. (B–D) Primary cultures from the duodenum/jejunum (WT and KO: *n *= 9 wells from 3 mice each) (B), ileum (WT and KO: *n *= 12–15 wells from 3 mice each) (C), or colon (WT: *n *= 22 wells from 7 mice. KO: *n* = 14–16 wells from 5 mice) (D) of WT (*Cre*-negative/*Gpr119*^fl^, white bars) and *Gpr119* KO (*Cre*-positive/*Gpr119*^fl^, grey bars) were assessed for GPR119-dependent GLP-1 release as in A above. Bars represent means + SEM. Significance was tested by one-way ANOVA, with post-hoc Bonferroni tests comparing (i) test agents vs basal control for the corresponding genotype (*p < 0.05, ***p < 0.001), and (ii) WT vs KO for each condition († *p *<0 .05, ††† *p *< 0.001).

Colon cultures from *Cre*-negative/*Gpr119*^fl^ and *Cre*-positive/*Gpr119*^wt^ mice were treated with 10 μM forskolin plus 100 μmol/l 3-isobutyl-1-methylxanthine (IBMX) to raise cAMP, the small molecule GPR119 agonist AR231453 (100 nmol/l) [19], 200 μmol/l 2-oleoylglycerol (2-OG), or 10 μmol/l oleoylethanolamide (OEA). No difference in secretion was seen between these genotypes, indicating that neither the Cre-allele nor the *Gpr119^fl^* allele alone altered GLP-1 release (Fig. 2A).

The same ligands were then applied to cultures from *Gpr119*-KO mice (*Cre*-positive/*Gpr119^fl^*) and *Cre*-negative/*Gpr119^fl^* mice (henceforth called WT). Secretion was measured separately from the colon, ileum, and duodenum/jejunum (Fig. 2B–D). AR231453 significantly increased GLP-1 release 4.6-fold from the colon and 2.9-fold from the ileum of WT mice; OEA significantly enhanced GLP-1 release by 3.9-fold in the colon and 2.1-fold in the duodenum/jejunum; 2-OG only increased secretion significantly in the colon (2.1-fold). Secretory responses to all three GPR119 ligands were significantly impaired in colonic cultures from *Gpr119*-KO mice (Fig. 2D). In ileal cultures, the response to AR231453 was reduced in *Gpr119*-KO tissue (Fig. 2C), whereas in duodenal/jejunal cultures, the enhanced secretion triggered by OEA was not impaired by *Gpr119*-KO (Fig. 2B).

### 3.3 cAMP imaging in primary cultured L-cells

**Fig. 3:**
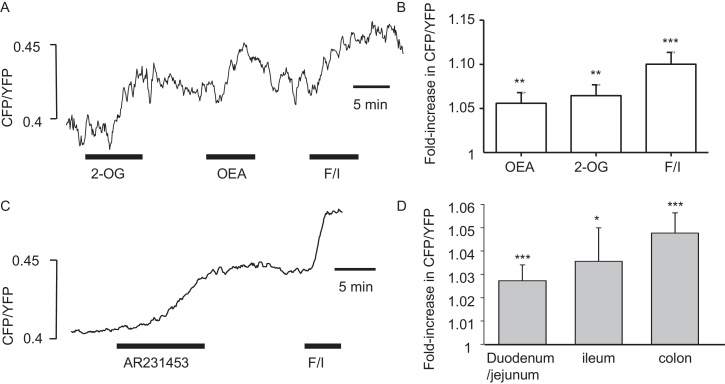
GPR119 agonists trigger cAMP elevation in primary L-cells. cAMP imaging in primary tissue cultures of GLU-Epac21 mice. (A) Sample trace showing representative colonic L-cell cAMP response to 2-OG (200 μmol/l), OEA (10 μmol/l) and forskolin + IBMX (F/I, 10 μmol/l each), added as indicated by the horizontal bars. cAMP was monitored as the CFP/YFP fluorescence ratio of the FRET sensor Epac2camps, expressed specifically in the L-cell population. (B) Mean (+SEM) FRET responses of 6 colonic L-cells to agonists applied as in A. (C) Sample trace showing representative colonic L-cell cAMP response to AR231453 (100 nmol/l) and F/I. (D) Mean (+SEM) responses to AR231453, measured as in C, for all L-cells tested from the duodenum/jejunum (*n *= 26), ileum (*n *= 11) and colon (*n *= 21). *p < 0.05, ***p < 0.001 vs basal, by one-sample Student’s *t*-test.

cAMP concentrations in primary L-cells were imaged in primary cultures from mice expressing a FRET-based cAMP sensor in proglucagon-expressing cells. In colonic cultures, 2-OG (200 μmol/l), OEA (10 μmol/l) and 100 nmol/l AR231453 triggered elevations of L-cell cAMP (Fig. 3). Particularly in the upper intestine, we observed that not all cells responded to test agents, and cells were allocated as responders if they showed a change of the FRET signal of >2% above baseline. In the duodenum 50% of L-cells (13 out of 26) exhibited cAMP responses to AR231453, compared with 45% (5/11) of L-cells in the ileum and 71% (15/21) in the colon. The mean amplitude of the cAMP response to AR231453 was not significantly different across intestinal tissues (Fig. 3D).

## 4 Discussion

Following the de-orphanization of GPR119, small molecules targeting this receptor were developed as potential new treatments for diabetes that would increase secretion from intestinal L-cells [25]. Although subsequent trials have not yet demonstrated that metabolic improvements can be brought about by the use of GPR119 agonists in humans with type 2 diabetes [20], there is still a high level of academic and commercial interest in GPR119 as a potential drug target [26], [27]. Our results show that L-cell GPR119 is a critical component of the sensing mechanism responsible for GLP-1 responses to ingested lipid, and that L-cells in the distal intestine respond to GPR119 agonists with elevated cAMP and GLP-1 secretion.

We show here that GPR119 ligands increase GLP-1 release from primary cultured ileal and colonic L-cells in a GPR119-dependent manner. Of the three GPR119 agonists tested, OEA and AR231453 were more effective than 2-OG. The magnitude of the secretory response triggered by the different GPR119 ligands increased progressively from the upper small intestine to the colon. Indeed, L-cell knockout of *Gpr119* largely abolished responses to OEA, 2-OG and AR231453 in the colon. In the ileum, where the secretory response was smaller, only OEA and AR231453 raised secretion in WT tissues above that found in the *Gpr119*-KO, and in the duodenum/jejunum, none of the ligands had a greater effect in WT than KO cultures. While our results suggest that the small response to OEA in the duodenum/jejunum of WT tissue is independent of GPR119, we cannot exclude the possibility that the proportion of L-cells undergoing Cre-dependent GPR119 excision differed between tissues and that more residual L-cells expressed GPR119 in the upper intestine. Arguing against this idea, however, AR231453 had little effect on GLP-1 secretion in the WT duodenum/jejunum, and OEA has been reported to activate other pathways such as PPARα that might influence GLP-1 secretion even in the absence of *Gpr119* [28].

The GLU-Epac transgenic mouse enabled us to monitor cAMP responses to GPR119 ligands in individual primary cultured L-cells. Not all L-cells were found to be responsive to AR231453, suggesting there may be a subpopulation of L-cells that do not express functional GPR119. There was a tendency for smaller and less frequent cAMP responses to AR231453 in the small intestine compared with the colon, although this did not reach statistical significance. These results do, however, mirror the gradient of GLP-1 secretory responses in cultures from the different regions. In line with these findings, we also reported previously that *Gpr119* expression appeared higher in colonic than small intestinal L-cells by qRT-PCR [23].

Mice with targeted deletion of *Gpr119* in L-cells exhibited a marked reduction of plasma GLP-1 levels after gastric oil gavage. This suggests that GPR119-dependent detection of luminally-generated 2-monoacylglycerols or locally-released OEA plays a major role in the post-prandial GLP-1 secretory response to orally ingested triglycerides. While long chain free fatty acids are also released during the luminal digestion of corn and olive oils, and are sensed by GPR119-independent pathways, likely involving GPR40 and GPR120 [4], our findings suggest that these pathways play a relatively minor role compared with GPR119 in mediating the GLP-1 secretory response to oral lipids. While our data support the development of GPR119 agonists to enhance GLP-1 secretion, the role of different intestinal regions in post-prandial physiology and as drug targets deserves further attention.
